# Expression of Bt Protein in Transgenic Bt Cotton Plants and Ecological Fitness of These Plants in Different Habitats

**DOI:** 10.3389/fpls.2020.01209

**Published:** 2020-08-07

**Authors:** Laipan Liu, Ruqing Guo, Qin Qin, Jianmei Fu, Biao Liu

**Affiliations:** ^1^ Key Laboratory on Biosafety of Nanjing Institute of Environmental Sciences, Ministry of Ecology and Environment, Nanjing, China; ^2^ State Environmental Protection Scientific Observation and Research Station for Ecology and Environment of Wuyi Mountains, Nanjing Institute of Environmental Sciences, Ministry of Ecology and Environment, Nanjing, China

**Keywords:** cotton, Cry1Ab/c, genetic engineering (GE), growth, field trial

## Abstract

Fitness is one of the key parameters to evaluate the effects of transgenic plants on the ecological environment. To evaluate the ecological risk of transgenic Bt cotton plants growing in different habitats, we determined the expression of the exogenous Bt gene and the fitness of transgenic and non-transgenic cotton plants in three habitats (farmland, grassland, and shrub). We observed that the expression of Bt protein in the farmland was significantly higher than that in the natural habitat, and when the growth environment was suitable, the Bt protein expression level showed a downward trend with the advancement of the growth. There were no significant differences in plant height, aboveground biomass, and seed yield between the Bt transgenic and non-transgenic cotton plants at the same growth stage under the same habitat. Nevertheless, in different habitats, the fitness of the same cotton line showed significant differences. In the farmland habitat, the plant height, aboveground biomass, and seed yield of both transgenic cotton and its non-transgenic isoline were significantly higher than that in the other two natural habitats. The results indicate that the expression of Bt protein does not increase the fitness of the parent plants and would not cause the weeding of the recipient cotton plants.

## Introduction

With the rapid development of resistance to chemical pesticides in cotton pests, such as *Helicoverpa armigera*, the production cost of cotton is increasing and consequently the income of farmers is getting lower (Lu et al., 2012). People are eager to find an effective solution to the problem of pesticide resistance in cotton pests. *Bacillus thuringiensis* (Bt) is a biological insecticide widely used in pest control in agriculture and forestry (Schnepf et al., 1998). In 1996, Bt-Cry1Ac transgenic insect-resistant cotton (Bt cotton) began to be commercialized in the United States (Bollgard I) and Australia (Ingard I), and has been widely planted in the world’s major cotton-producing countries. In 2018, the planting area of Bt cotton in the world reached 24.9 million hectares (ISSAA, 2019). The commercial planting of transgenic cotton effectively reducing the cost of cotton production, increasing the cotton production, and generating huge economic benefits for the farmers (Naseem and Qaim, 2016). With increasing number of genetically modified (GM) crops entering commercial production, determination of their long-term fitness effects in natural habitats has become an important part of safety assessment and monitoring of such crops (Burke and Rieseberg, 2003; Parimala and Muthuchelian, 2010; Arpaia et al., 2014; Sankaranarayanan and Nalayini, 2015; Lu et al., 2016; Jin et al., 2017; Koller et al., 2019).

On the one hand, plants with exogenous genes can be endowed with resistance to stress to improve their fitness, so that they can survive and reproduce better under adverse environmental conditions. On the other hand, without adverse environmental conditions, the expression of exogenous genes may incur a cost of fitness because of the occupancy of energy and material resources. If the available resources of plants are fixed, the energy consumed for the expression of exogenous genes will inevitably lead to a decrease in resources for growth and reproduction, resulting in the lower growth and reproduction capacity of transgenic plants compared to that of non-transgenic plants (Filipecki and Malepszy, 2006; Xia et al., 2009; Yang et al., 2015). Under conditions of low or no selective pressure, GM plants may show the cost of fitness, which to some extent reduces the possibility of exogenous genes escaping from non-GM plants (Zhang et al., 2012). If the fitness cost is small or there is no fitness cost, then the transferred genes may be preserved and spread in non-transgenic or wild weed populations; this means that there is a risk of exogenous genes escaping to non-transgenic plants (Ellstrand, 2003). Therefore, ecological fitness study is an important parameter to evaluate the ecological effect of transgenic plants. The influence of exogenous genes on the fitness of recipient plants is affected by multiple factors, including the type and construction methods of exogenous genes, the characteristics of transgenic plants, genetic background, the existence of selective pressure, and the environmental conditions under which the transgenic plants grow (Labra et al., 2001; Noro et al., 2007; Mahmoodurrahman et al., 2012). With the large-scale and long-term planting of GM crops, the possibility of these crops entering the natural habitat is greatly increased, and it is increasingly possible that exogenous genes in transgenic plants can enter the wild close relatives of their natural habitats through gene drift (Ellstrand and Hoffman., 1990; Snow et al., 2005; Lu and Yang 2009; Ellstrand et al., 2013). Whether transgenic plants entering the natural habitat and wild related species containing exogenous genes can survive or expand their populations is a scientific question that must be answered in the environmental safety evaluation and monitoring of transgenic organisms.

Transgenic Bt cotton is the predominant commercial transgenic crop in China that has been cultivated for the longest period of time (Lu et al., 2012). However, few studies have been conducted on assessing the fitness of GM cotton in some wild or semi-wild environments. Whether transgenic insect-resistant cotton can survive and proliferate for a long time, whether the insertion of exogenous genes affects the suitability of the recipient cotton for poor ecological environment and then causes exogenous gene escape and invasion, and whether there is a risk of weeding have not been thoroughly studied. We performed experiments to evaluate the ecological fitness effects of transgenic cotton Bt under natural conditions facing different environmental. The results will provide new clues and scientific basis for evaluating the impact of Bt expression on plant fitness. After GM crops escape from their field habitat and enter the natural habitat, the expression of exogenous genes in the natural habitat directly determines the performance and ecological consequences of GM crops in natural habitat. Moreover, the expression of exogenous Bt genes under different environmental conditions was studied to assess the ecological risk of Bt-transgenic plants entering the natural habitat after escape.

## Materials and Methods

### Cotton Varieties

The cotton varieties used in this study were transgenic Cry1Ab/c cotton Zhong30 and its non-transgenic isolines, Zhong16, which were provided by the Cotton Research Institute of Chinese Academy of Agricultural Sciences. The transgene has one insertion and used CaMV 35S for transgene expression in Zhong30. All cotton plants were transplanted to a field after cultivation in a greenhouse.

### Experimental Design

In 2014 and 2015, three experimental sites were set up in Hengxi, Jiangning District, Nanjing, China, which had three different habitats (farmland, grassland, and shrub habitats, respectively) with significant differences in physicochemical properties of the soil (**Table 2**). The linear distances among the three habitats did not exceed 500 m. Cotton row spacing was 1 m, plant spacing was 1.5 m. Zhong 16 was planted in the East and Zhong 30 in the West. A total of 200 cotton plants are planted on each experimental site. During the survey, samples were randomly divided into groups, for details, see the survey methods for each index below. The composition and biomass of weeds were analyzed, and the physical and chemical properties of soil were analyzed by soil sampling.

### Weed Species and Biomass

Five squares (50 cm × 50 cm) were randomly selected at each experimental site. All the plants in the squares were cut at ground level and packed in nylon mesh bags. Under the condition of direct sunlight, the weeds were dried to a constant mass in the ventilated area, and the mass of weeds was recorded. The composition and biomass of weeds are shown in **Table 1**.

**Table 1:** Weed composition and management practices at the different experimental sites.

Experimental site	Ecological type	Weed species and biomass	Farm management
I	Field habitat	None	Normal field irrigation, fertilization, weeding, and no pesticide application
II	Grassland habitat	*Pennisetum alopecuroides* (L.) Spreng. (589.08 g/m^2^), *Artemisia lavandulaefolia* DC. (15.82 g/m^2^), *Solidago canadensis* L. (1.4 g/m^2^), *Lonicera japonica* Thunb. (1.72 g/m^2^), *Mosla dianthera* (Buch.-Ham. ex Roxburgh) Maxim. (1.27 g/m^2^)	No fertilizer; irrigation for several days after transplanting ensured survival of cotton seedlings, without any other management.
III	Shrub habitat	*Conyza sumatrensis* (Retz.) Walker (1.42 g/m^2^), *Imperata cylindrica* (Linn.) Beauv. (1.27 g/m^2^)	No fertilizer; irrigation for several days after transplanting ensured survival of cotton seedlings, without any other management.

### Physical and Chemical Properties of the Soil

Five samples were taken from each cotton variety in each experimental site, and the five sampling sites formed an S shape. Clean up the sundries 20 mm deep on the surface and cut off a soil sample 5- to 7-cm thick with a spade. The thickness of soil sample at each point was almost the same. After natural air drying, grinding, and sieving, the samples were sent to Nanjing Institute of Soil Research, Chinese Academy of Sciences for determination of the physical and chemical properties according to Agricultural Standard of People’s Republic of China NY/T 1121.7-2014. The specific conditions at each experimental site are shown in **Table 2**.

**Table 2:** Physicochemical properties of the soil at different experimental fields (mean ± stdev).

Ecological type	pH	Organic matter (g/kg)	Total nitrogen (g/kg)	Total phosphorus (g/kg)	Total potassium (g/kg)	Available phosphorus (mg/kg)	Available potassium (mg/kg)
Field habitat	7.14 ± 0.16^a^	31.25 ± 2.11^a^	1.51 ± 0.25^a^	1.74 ± 0.21^a^	14.27 ± 1.64^a^	13.78 ± 1.61^a^	129.45 ± 9.80^c^
Grassland habitat	5.50 ± 0.33^b^	33.73 ± 2.75^a^	1.37 ± 0.14^a^	1.17 ± 0.35^b^	10.72 ± 1.78^b^	9.86 ± 0.88^b^	165.94 ± 6.13^a^
Shrub habitat	5.71 ± 0.34^b^	9.72 ± 1.70^b^	0.71 ± 0.30^b^	1.20 ± 0.31^b^	11.70 ± 0.99^b^	6.60 ± 0.79^c^	147.90 ± 8.12^b^

Data followed by the same lowercase letters in the same column are not significantly different at 0.05 level.

### Investigation of Insects and Diseases

Five investigation sites were set up in the four corners and the middle position of zhong30 and zhong16 respectively, and 10 cotton plants were investigated in each investigation site. Three times of investigation of insects and diseases were conducted by direct observation method at the seedling stage, flowering stage and boll opening stage of cotton.

The species and number of insects on each cotton plant and the degree to which cotton leaves were eaten by insects were investigated and recorded. The damage degree is divided into 6 levels: 0. no feeding; 1. the damage is needle-like; 2. the damage area is less than 1/4; 3. the damage area is less than 1/2; 4. the damage area is more than 1/2; 5. the leaves were completely eaten. Record the incidence of *Fusarium* and *Verticillium wilts* of cotton.

### Quantification of Cry1Ab/c Protein by Enzyme-Linked Immunosorbent Assay

Two hundred cotton plants were divided into 3 groups at each experimental site and 10 cotton plants were randomly selected from each group for marking for each cotton variety. Cotton was sampled at the bud, boll, and boll opening stages. A complete leaf from the top of each plant was collected. The leaf was immediately placed in a 10-ml sealed centrifuge tube after being detached and stored in a liquid nitrogen tank. After reaching the laboratory, the samples were stored in a deep-freezer at −70°C. The expression of Cry1Ab/c protein in Zhong30 cotton was quantified using a QualiPlate Kit for Cry1Ab/Cry1Ac (EnviroLogix Inc., Portland, ME, USA). About 20 mg of tissue sample was weighed and its exact mass was recorded. The test was done according to the manufacturer’s instructions. The optical density (OD) was measured at 450 nm using a microplate reader (Infinite M2000; Tecan Group Inc., Männedorf, Switzerland). The calibration was done by generating a standard curve of OD against the protein content using the following concentrations of the Bt protein (Cry1Ab) standard (EnviroLogix Inc.): 0.03125, 0.0625, 0.125, 0.25, 0.5 ng, 1.0, 2.0, and 4.0 ng mL^−1^. The level of Cry1Ab/c protein in the fresh cotton leaf samples was determined using the standard curve and the dilution ratios of the extract (μg g^−1^ FW).

### Measurement of the Fitness Indices of Vegetative and Reproductive Growth

One hundred cotton plants were divided into 3 groups in each cotton variety at each experimental site and 10 cotton plants were randomly selected and marked from each group. The height of 30 randomly marked cotton from soil to terminal tip was measured (accurate to centimeter) using a measuring tape. To determine the aboveground biomass, five unmarked cotton plants were randomly selected from two kinds of cotton at each site. The plant was cut off from a position nearest to the surface, dried under natural shade to a constant mass, and then weighed using a balance (PB602-N, Mettler Toledo).

To detect the effect on the reproductive growth, seeds of marking cotton were harvested. The seeds were dried and cotton was removed from around their short nap with a portable cotton clothes sorting mill (mj57-110d, Xinxing Cotton Machinery Factory, Dong-guang county, Hebei province, China). All the cottonseeds were counted and weighed using an electronic balance (tp-214, Denver Instruments Co. Ltd.), accurate to 0.01 g.

### Data Analysis

The expression of the exogenous Cry1Ab/c protein in the Zhong30 cotton plants might be affected by the growth stage and growth environment. Therefore, Tukey’s HSD test with the alpha level at 0.05 was used to determine the difference of exogenous Cry1Ab/c protein expression in different growth stages of cotton in different experimental sites.

In this study, two-factor (factor 1 is cotton variety, factor 2 is experimental site) was used to compare the difference of fitness index between zhong16 and zhong30 cotton. Test factor 1, the relative fitness was calculated by dividing the fitness index of zhong30 cotton by the fitness index of zhong16 cotton, and the relative fitness of each index is added to take the mean value to calculate the comprehensive fitness index. Test factor 2, with farmland ecosystem experimental site I as control, each fitness index of cotton in other experimental sites was divided by corresponding fitness index of experimental site I cotton, and the relative fitness of each index is added to take the mean value to calculate the comprehensive fitness index. Tukey’s HSD test was performed to determine the significant differences of the fitness.

All statistical analyses were performed using SPSS v. 20.0 for Windows (IBM Corp., Armonk, NY, USA).

## Results

### Insects and Diseases in Cotton Fields

Based on the investigation of cotton diseases and insect pests, the results showed that because the experimental area was located in the non-cotton area and there was no cotton planting in the surrounding kilometers, there were almost no common cotton diseases in the experimental area. Only a small number of neutral insects, such as *Araneida*, *Coccinellidae*, and *Locusta migratoria manilensis* (Meyen) were found at the three experimental sites. Cotton pests such as *Helicoverpa armigera*, *Aphis gossypii* Glover and *Apolygus lucorμm* (Meyer-Dür.) have hardly been found. The target insects of transgenic cotton with *Bt* gene are *Lepidoptera* insects, so it can be determined that our experimental sites are in a state of low insect pressure.

### Bt Expression in Transgenic Lines Under Different Habitats

In the two-year survey, the trend of change in the content of Bt protein in the leaves of transgenic Bt cotton plants was different at different growth periods at the same experimental site (**Table 3**). The protein content in the plants at experimental site I and experimental site II decreased gradually with the development of the plant, but the protein content in the plants at experimental site III did not show the same trend. During the three growth stages of the leaves, the content of Bt protein at experimental site I was significantly higher than at the other experimental sites (p< 0.05). The content of Bt protein at experimental site II was higher than that at experimental site III. There were significant differences in the content at the bud stage and flowering and boll-forming stages (p< 0.05), but no significant differences were observed in the content at the boll-opening stage (p> 0.05) (**Table 3**).

**Table 3:** Content of Bt protein in the leaves of Zhong30 plants at different experiment sites during the different growth stages (μg/g fresh mass) (mean ± stdev).

Year	Growth stage	Field habitat	Grassland habitat	Shrub habitat
2014	Bud stage	4.92 ± 0.31^a^	3.20 ± 1.36^bc^	0.46 ± 0.44^e^
	Flowering and boll-forming stage	4.14 ± 0.90^ab^	2.19 ± 0.98^cd^	0.76 ± 0.28^de^
	Boll-opening stage	3.52 ± 0.92^abc^	1.23 ± 0.72^de^	0.78 ± 0.51^de^
2015	Bud stage	4.72 ± 1.01^a^	3.51 ± 1.25^ab^	0.37 ± 0.32^d^
	Flowering and boll-forming stage	4.04 ± 0.96^ab^	2.49 ± 0.94^bc^	0.85 ± 0.22^d^
	Boll-opening stage	3.82 ± 0.83^ab^	1.23 ± 0.44^cd^	0.78 ± 0.27^d^

Data followed by the same lowercase letters in the same year are not significantly different at 0.05 level, n = 30.

### Plant Height of Transgenic and Conventional Cotton Under the Three Habitats

**Figure 1:**
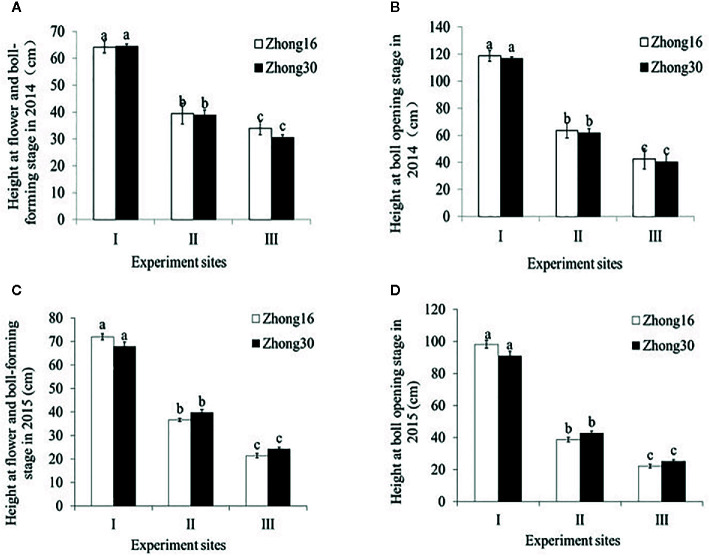
Plant height (mean ± SEM) of Zhong30 and Zhong16 cotton plants grown under field (I), grassland (II), and shrub (III) habitat at two stages in 2014 and 2015: **(A)** Flowering and boll-forming stage in 2014, **(B)** boll-opening stage in 2014, **(C)** flowering and boll-forming stage in 2015, **(D)** boll-opening stage in 2015. Data followed by the same lowercase letters are not significantly different at 0.05 level.

As shown in **Figure 1**, there were no significant differences in the height between Zhong30 and Zhong16 plants under the same biotope at the same stage in 2014 and 2015 (p>0.05). The height of the same kind of cotton plants was significantly different at the different experimental sites during all the survey periods (p<0.0001) (**Figure 1**). The height of cotton plants at experimental site I was significantly higher than the height at the other experimental sites with the natural system (p<0.0001). Interestingly, significant difference in heights was observed between the two experimental sites with the natural habitat; the height at experimental site II was significantly higher than that at experimental site III (p<0.0001).

### Aboveground Biomass of Transgenic and Conventional Cotton Under Three Habitats

**Figure 2:**
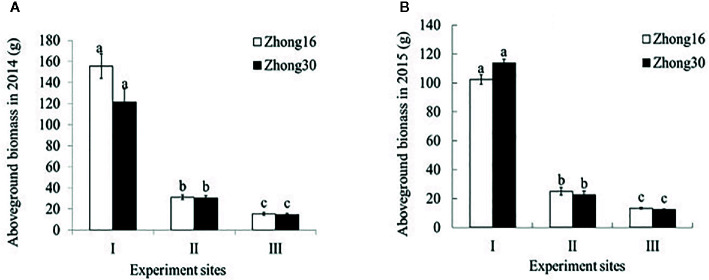
Aboveground biomass (mean ± SEM) of Zhong30 and Zhong16 cotton plants grown under field (I), grassland (II), and shrub (III) habitat in 2014 **(A)** and 2015 **(B)**. Data followed by the same lowercase letters are not significantly different at 0.05 level.

As shown in **Figure 2**, there were no significant differences in the aboveground biomass between Zhong30 and Zhong16 under the same biotope in 2014 and 2015 (p>0.05). The aboveground biomass of the same cotton line was significantly higher for those grown under the field habitat (experimental site I) than for those grown under the natural conditions (experimental site II and III) by about 4.0 to 10.1-fold in 2014 and 2015. The biomass of the cotton plants grown under the grassland habitat (experimental site II) was 1.7 to 2.0-fold higher than of those grown under the shrub habitat (experimental site III).

### Reproductive Growth of Transgenic and Conventional Cotton Under Different Biotopes

In 2014, the seed mass per plant of Bt cotton was less than that of non-Bt cotton, but the difference was not significant at experimental site I (**Table 4**). Only one Zhong 16 cotton produced seed in experimental site II and experimental site III, which was not enough for statistical analysis. In 2015, the seed mass per plant of Zhong 30 was greater than that of Zhong 16, but the difference was not significant (p=0.0827). There were only two Zhong 30 cotton plants producing bolls at experimental site III, and no seeds were found at experimental site II, and therefore, no statistical analysis could be done.

**Table 4:** Production of seeds per plant of different cotton varieties at different experimental sites in 2014 and 2015 (g) (mean ± stdev).

Year	Variety	Field habitat	Grassland habitat	Shrub habitat
2014	Zhong16	9.91 ± 3.3^a^	0.27	0.17
	Zhong30	7.81 ± 0.78^a^	0.36 ± 0.05	0.18 ± 0.01
2015	Zhong16	8.38 ± 2.33^a^	0	0
	Zhong30	9.57 ± 1.98^a^	0	0.16 ± 0.04

Data followed by the same lowercase letters in the same column in the same year are not significantly different at 0.05 level, n = 30.

### Comparison of the Fitness of Transgenic and Conventional Cotton Plants Under Different Biotopes

**Figure 3:**
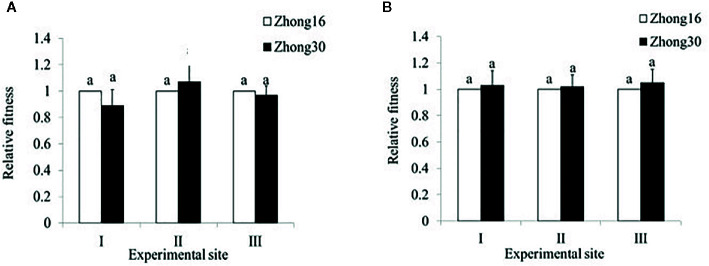
The overall fitness of transgenic Bt cotton Zhong30 relative to that of non-transgenic cotton Zhong16 grown under field (I), grassland (II), and shrub (III) habitat in 2014 **(A)** and 2015 **(B)**. Data followed by the same lowercase letters are not significantly different at 0.05 level.

**Figure 4:**
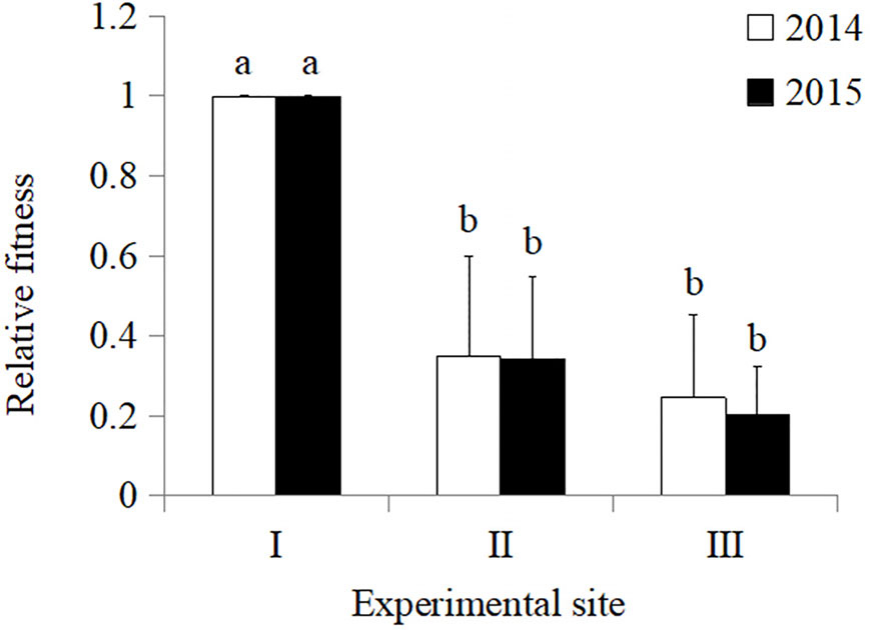
The overall fitness of cotton in grassland (II), and shrub (III) habitat relative to that of cotton in field (I) habitat in 2014 and 2015. Data followed by the same lowercase letters are not significantly different at 0.05 level.

In 2014, the total fitness of Zhong30 relative to that of Zhong16 at experimental site II was 1.07, indicating that Zhong30 showed fitness benefits in grassland habitat; the total fitness of Zhong30 relative to that of Zhong16 at experimental site I and experimental site III was 0.89 and 0.97, respectively, indicating that Zhong30 showed a certain fitness cost in field and shrub habitats. In 2015, the total fitness of Zhong30 relative to that of Zhong16 at experimental sites I, II, and III was 1.03, 1.02, and 1.05, respectively (**Figure 3**). Both in 2014 and 2015, compared with cotton in experimental site I, cotton in experimental site II and III showed significant fitness cost (**Figure 4**). The results showed that cotton growth environment rather than expression of Bt protein had more significant effect on cotton fitness.

## Discussion

There is an important relationship between the fitness of transgenic crops and the plant growth environment (Burke and Rieseberg, 2003). The purpose of this experiment was to simulate the changes of Bt protein expression and fitness of transgenic cotton after escaping to the natural ecosystem, so the selected experimental site and its surroundings had no cotton planting for many years. The cotton plants were disease-free and under the condition of no pest pressure or low pest pressure, which could better simulate the natural ecosystem such as grassland and shrub habitat. Experimental site I belongs to field habitat. The content of soil organic matter is high, and the content of mineral elements needed for the growth of all kinds of plants is closest to the level of ordinary farmland. In the process of cotton growth, weeding, fertilization and irrigation are carried out according to the way of farmland management, but no pesticides are applied. Experimental site II belongs to grassland habitat, the content of organic matter is high, but the content of other mineral elements is low, and the stress of weeds in experimental site II is very serious. Experimental site III belongs to shrub habitat. There are few weeds, but the soil fertility is very low, soil erosion is serious, and cotton is vulnerable to drought stress. After transplanting, cottons in experimental sites II and three are allowed to grow freely without any artificial management.

The properties of soil were reported to affect the expression of Bt protein; drought or saline soil conditions could affect the growth and survival of plants, thereby, affecting the expression of Bt protein (Rochester, 2006). Soil water deficit resulted in the decline of the insecticidal protein expression in bolls of Sikang 3, a hybrid cultivar cotton transgenically expressing a *Bt* gene (Zhang et al., 2017). Under high and low temperature, drought, and flooding stress habitats, the expression of the exogenous Bt protein in transgenic maize was significantly lower than that under normal habitats (Trtikova et al., 2015). At the same time, the expression of exogenous Bt protein in transgenic rice in habitats stressed with NaCl, flooding (Luo et al., 2008), low, medium, and high salinity (Luo et al., 2017; Fu et al., 2018) showed a decreasing trend compared to the expression in normal habitats. In our study, we compared the content of Bt protein in transgenic cotton plants at different growth stages under different environments. The results for the two years showed that the content of Bt protein in the leaves of Bt transgenic cotton plants in field habitat was significantly higher than that in the other habitats. This may be because under adverse conditions, transgenic plants will produce more substances to resist adverse survival factors (for example, under drought conditions, transgenic plants will produce more drought-resistant and high-temperature resistant proteins), thus affecting the expression of foreign genes. The abovementioned results showed that the external environment exerts a great effect on the expression of the exogenous Bt protein.

Cotton plants were reported to be most resistant to insects in the early stages of their growth as the concentration of Bt toxin declined during the growing season (Sivasupramaniam et al., 2008; Brévault et al., 2013). Cot102 insect-resistant cotton exhibited the best resistance to *H. armigera* in the early growth stage; with the development of insect-resistant plants, the resistance to phytophagous insects showed a decreasing trend (Llewellyn et al., 2007). The concentration of Bt protein in the terminal leaves declined significantly during the growing season, with the mean toxin concentration being 9.2-times higher for Cry1Ac and 2.9-times higher for Cry2Ab in the young (squaring) cotton than in the old (fruiting) cotton (Carrière et al., 2019). In 2014 and 2015, with the development of Bt transgenic cotton plants, the concentration of Bt protein in field and grassland habitats decreased gradually, while the concentration of Bt protein in shrub habitats remained at a low level with no uniform trend. The soil of cotton in shrub habitat is barren and vulnerable to drought stress, which may be the reason why the Bt protein content of Bt transgenic cotton in shrub habitat is the lowest among the three habitats and does not show significant change in cotton growing season.

The existence of exogenous genes did not change the fitness effect of transgenic cotton plants in 12 natural habitats in Australia, and therefore, the ecological risk of Bt transgenic cotton was inferred to be low (Eastick and Hearnden, 2006). Under low insect pressure, the seed yield of transgenic insect-resistant rice was lower than that of non-transgenic rice, but the difference was not significant (Yang et al., 2011). The growth and reproductive capacity of Cry1Ab/c transgenic rice (Su et al., 2013) was similar to those of their parents under a semi-wild growth environment (high and low insect pressure), which indicated that Cry1Ab/c transgenic rice had lower ecological risk under the semi-wild growth environment. The comparative study on Cry1Ab/c transgenic rice, HH1, and its parent rice, MH63, in Nanchang (having a natural ecosystem) showed that the reproductive and competitive abilities of HH1 and MH63 rice were similar in the natural ecosystem with high insect pressure. (Liu et al., 2015). In this study, in the agroecosystem (field habitat (I)), the growth process of cotton was carefully managed by humans, and there was sufficient water and fertilizer. The growth and reproduction indices (height, aboveground biomass, and seed production per plant) of cotton plants at this site were significantly higher than those for cotton plants of the same kind but grown at other sites. Both the natural ecosystems (grassland (II) and shrub (III) habitats) are subjected to severe environmental stress and almost no seeds were produced in the Bt transgenic and non-transgenic cotton plants. There was no significant difference in the relative fitness between Zhong30 and Zhong16 grown in the same habitat. According to the results of our research, there is no significant difference in the fitness between the Bt transgenic cotton and conventional cotton plants grown under insect-free or low insect pressure conditions in the field ecosystem and in the natural environment under drought and weed stress conditions.

However, the insertion of exogenous genes results in the fitness cost of the host reproductive indicators. In saline-alkaline soils, the insect-resistant transgenic Cry1C* rice T1C-19 showed a strong reproductive capacity, and significantly reduced the loss of yield caused by insects, therefore leading to a higher yield than that of its non-transgenic counterparts MH63 grown (Fu and Liu, 2020). The vegetative and reproductive growth abilities showed a significantly higher fitness cost for the Cry1Ab/c transgenic rice, Huahui1, than that for the parental rice, Minghui63, grown under the same normal farmland and saline-alkaline soil conditions (Fu et al., 2018). Under the same drought conditions, the yield of GM insect-resistant cotton, RCHB 708, was significantly higher than that of non-GM cotton, LRA 5166, and GM cotton showed a fitness advantage (Sankaranarayanan and Nalayini, 2015). Under low insect pressure, Bt/CpT1 transgenic rice showed potential fitness cost in the yield traits (yield per plant, number of grains per plant, number of effective panicles, etc.) compared to that for the parent rice (Xia et al., 2010). The yield (Kg/100 hill) of Bt-SY63 Cry1Ab/c transgenic rice was lower than that of its parent rice, SY63, under low insect pressure with the application of chemical insecticides for two years (Wang et al., 2010). The 1000-grain mass of the insect-resistant rice HH1 was significantly lower than that of its parent rice MH63 under low insect pressure (Xia et al., 2011). In this experiment, the growth of cotton plants was very unsatisfactory due to the environmental stress at the experimental sites II and III. The yield of both the GM and non-GM cotton seeds was low and even zero for some plants.

## Conclusion

The expression of exogenous genes in transgenic plants is crucial to the suitability of such plants. We detected the expression of exogenous Bt gene in Bt transgenic cotton plants grown under different environments, and found that the expression levels decreased significantly under the natural environment in the presence of different environmental factors. Therefore, we speculate that under the stress imposed by environmental factors, if the exogenous Bt gene escapes into a natural environment with low insect pressure, the expression behavior of the exogenous Bt gene would be affected by environmental factors, such as drought and weed stress; the fitness benefit of the exogenous Bt gene would not be observed in such cases, and no competitive advantage would be achieved with respect to the species growing in the natural environment. In the present study, there were no significant differences in plant height, aboveground biomass, and seed yield between the Bt cotton and non-Bt cotton plants grown in the natural ecosystem under low insect pressure. Although cotton plants can grow in natural ecosystem, they have weak reproductive capacity and very low seed yield. If the exogenous Bt gene enters the natural ecosystem, it will not increase the fitness of the parental donors, and therefore, the exogenous Bt gene will not cause the weeding of the recipient cotton.

## Data Availability Statement

All datasets generated for this study are included in the article/supplementary material.

## Author Contributions

LL and RG are co-first authors of the article. LL analyzed the data and wrote the manuscript. RG and QQ carried out the experiments, JF helped analyze the data, and BL designed and helped to calibrate the manuscript.

## Funding

This work was supported by the “Basic Scientific Research Program in National Nonprofit Scientific Research Institutes (GYZX190103)”; “National Major Special Projects for Genetically Modified Organisms (2016ZX08012-005)”; “Jiangsu Planned Projects for Postdoctoral Research Funds (2018K167C)”.

## Conflict of Interest

The authors declare that the research was conducted in the absence of any commercial or financial relationships that could be construed as a potential conflict of interest.
